# Severe COVID-19 associated hyperglycemia is caused by beta cell dysfunction: a prospective cohort study

**DOI:** 10.1038/s41387-023-00241-7

**Published:** 2023-07-17

**Authors:** Jan Gojda, Kateřina Koudelková, Anna Ouřadová, Alexander Lang, Magdaléna Krbcová, Alexandra Gvozdeva, Viktor Šebo, Lotte Slagmolen, Jana Potočková, Petr Tůma, Lenka Rossmeislová, Michal Anděl, Fredrik Karpe, Sabrina Schlesinger

**Affiliations:** 1 Department of Internal Medicine, Third Faculty of Medicine, Charles University, and Královské Vinohrady University Hospital, Prague, Czech Republic; 2 Institute for Biometrics and Epidemiology, German Diabetes Center (Deutsches Diabetes-Zentrum/DDZ), Leibniz Center for Diabetes Research at Heinrich Heine University Düsseldorf, Düsseldorf, Germany; 3 German Center for Diabetes Research (DZD), Partner Düsseldorf, Düsseldorf, Germany; 4 Department of Pathophysiology, Third Faculty of Medicine, Charles University, Prague, Czech Republic; 5 Faculty of Movement and Rehabilitation Sciences, Katholieke Universiteit Leuven, Leuven, Belgium; 6 Department of Hygiene, Third Faculty of Medicine, Charles University, Prague, Czech Republic; 7 Oxford Center for Diabetes, Endocrinology, and Metabolism, University of Oxford, Oxford, UK

**Keywords:** Type 2 diabetes, Pre-diabetes

## Abstract

**Background:**

COVID-19, an infectious disease caused by SARS-CoV-2, was shown to be associated with an increased risk of new-onset diabetes. Mechanisms contributing to the development of hyperglycemia are still unclear. We aimed to study whether hyperglycemia is related to insulin resistance and/or beta cell dysfunction.

**Materials and methods:**

Survivors of severe COVID-19 but without a known history of diabetes were examined at baseline (T0) and after 3 (T3) and 6 (T6) months: corticosteroids use, indirect calorimetry, and OGTT. Insulin response and sensitivity (IS) were expressed as insulinogenic (IGI), disposition (DI), and Matsuda insulin sensitivity index (ISI). Resting energy expenditure (REE) and respiratory quotient (RQ) was calculated from the gas exchange and nitrogen losses.

**Results:**

26 patients (out of 37) with complete outcome data were included in the analysis (age ~59.0 years; BMI ~ 30.4, 35% women). Patients were hypermetabolic at T0 (30.3 ± 4.0 kcal/kg lean mass/day, ~120% predicted) but REE declined over 6 months (ΔT6-T0 mean dif. T6-T0 (95% CI): −5.4 (−6.8, −4.1) kcal/kg FFM/day, *p* < 0.0001). 17 patients at T0 and 13 patients at T6 had hyperglycemia. None of the patients had positive islet autoantibodies. Insulin sensitivity in T0 was similarly low in hyperglycemic (H) and normoglycemic patients (N) (T0 ISI_H_ = 3.12 ± 1.23, ISI_N_ = 3.47 ± 1.78, *p* = 0.44), whereas insulin response was lower in the H group (DI_H_ = 3.05 ± 1.79 vs DI_N_ = 8.40 ± 5.42, *p* = 0.003). Over 6 months ISI (ΔT6-T0 mean dif. T6-T0 for ISI (95% CI): 1.84 (0.45, 3.24), *p* = 0.01)) increased in the H group only.

**Conclusions:**

Patients with severe COVID-19 had increased REE and insulin resistance during the acute phase due to the infection and corticosteroid use, but these effects do not persist during the follow-up period. Only patients with insufficient insulin response developed hyperglycemia, indicating that beta cell dysfunction, rather than insulin resistance, was responsible for its occurrence.

## Background

COVID-19, an infectious respiratory disease caused by a novel severe acute respiratory syndrome coronavirus 2 (SARS CoV-2) [1] emerged in 2019 and has since spread throughout the world, causing an unprecedented pandemic [2]. Although the main symptoms of COVID-19 are related to the respiratory system, metabolic disturbances, hyperglycemia, and new-onset diabetes have been reported soon after the pandemic outbreak [3, 4]. Hyperglycemia was the most frequently described phenomenon that was associated with an increased risk of adverse outcomes (e.g., the severity of the disease, physiological stress and need for intubation) [5, 6], especially in patients with known diabetes [7]. Although elevated blood glucose levels are common during critical illness, the prevalence of hyperglycemia and new-onset diabetes in patients with COVID-19 infection appears to be unexpectedly high [8].

Since the first reports, intensive research has been conducted on possible causes of this phenomenon with many suggested mechanisms involved [5]. Abnormalities related to insulin resistance (IR) and beta cell dysfunction were suggested [9, 10]. IR is associated with hypermetabolism during the acute state of COVID-19 as a stress response and increased drive of counterregulatory hormones (such as catecholamines and cortisol) resulting in a decline in both skeletal muscle and liver insulin sensitivity [11]. Increased lipolysis and elevated circulating non-esterified fatty acids (NEFA) were also described [12]. Damaged mitochondria in peripheral tissues cannot meet the increased energetic demands caused by SARS-CoV-2-triggered inflammation, with a subsequent increase in reactive oxygen species (ROS) production [13]. Beta cells, on the other hand, can be affected by many suggested mechanisms: systemic and islet renin-angiotensin-aldosterone system (RAAS) activation, islet redox stress, systemic and islet inflammation, islet amyloid deposition, islet fibrosis, and/or failure due to apoptosis and capillary rarefaction [14]. Direct damage by replicating the virus in beta cells was also suggested [15]. The virus may also damage the endothelium, which further allows the impairment of the microvasculature and pericytes in the islets of Langerhans [16].

Retrospective studies that focused on the incidence of new-onset diabetes or hyperglycemia during the acute stage of the disease showed a wide distribution between 2.8 and 49.2% in the general population [17–20] and 35% to 85% in the critically ill [10, 21, 22]. The studies are mainly of retrospective design and methodologically very heterogeneous with different populations, end-point, and glycemic cut-offs. Moreover, it is not clear how long the observed changes persisted over time and whether insulin-glycemic indices tend to improve; limited evidence suggests they persist for up to two months [9].

This prospective cohort study aimed to investigate whether hyperglycemia persists in patients who survive severe COVID-19 over a follow-up period of 6 months and to explore possible causes of hyperglycemia, including insulin sensitivity/secretion, energy expenditure, substrate preference, and metabolic flexibility.

## Methods

### Subjects and design

The monocentric prospective cohort study COMETA (COVID-19 metabolic and nutritional consequences: prospective observational study) was conducted from March to November 2021 at the University Hospital Královské Vinohrady, Prague, Czech Republic. All consecutive patients aged over 18 years who were short-term survivors (i.e., weaned from artificial ventilation/ECMO/oxygen) were screened (March–April 2021) at specialized COVID-19 wards. Inclusion criteria were positive PCR/antigen nasopharyngeal test and severe course of the disease defined as bilateral pneumonia associated with COVID-19 verified by CT/CXR with respiratory failure defined as any need for oxygen support [23]. Exclusion criteria were known diabetes in medical history, chronic lung disease, active cancer, neurological disease with impaired mobility, acutely decompensated endocrine disease (thyroid, adrenal, etc.), and pregnancy in women. The patients were examined once they were weaned from oxygen support, not later than 4 weeks from the onset of the disease to capture the acute phase. In total, 37 patients met the eligibility criteria and were enrolled and examined (25th April–11th May 2021). Of these, five participants were excluded from the analysis due to extreme values in the outcome variables of interest at the initial visit (T0) (Figure S2). Patients were re-examined after 3 months (T3) and 6 months (T6), respectively. Six participants were lost to follow-up (not willing to participate, *n* = 6). Therefore, data from 26 participants with complete outcome data at follow-up were available for analysis. The STROBE flow chart is shown in Figure S1.

All participants signed informed consent prior to enrolment. The research protocol was approved by the Ethics Committee of University Hospital Kralovske Vinohrady (EK-VP-14-0-2021) and the study was conducted under GCP following the Declaration of Helsinki.

### Clinical examination

#### Anthropometry and medical examination

All examinations were performed after fasting overnight. Each subject underwent a basic medical examination with an anthropometric examination (height, weight, body mass index (BMI), waist circumference, and waist-to-hip ratio). Body composition was determined by bioimpedance analysis (BIA, Quadscan, Bodystat, UK) and expressed as skeletal muscle mass, active tissue mass (ATM, i.e., fat-free mass), and fat mass in kg and percentages respectively. Each participant filled out a questionnaire on symptoms related to COVID-19 under the guidance of a physician and a Baecke questionnaire to assess habitual physical activity [24]. Data on inpatient course (i.e., date of admission, ventilatory support, corticosteroids, etc.) were derived from available medical records.

#### Blood sampling and laboratory analysis

A peripheral venous blood sample was drawn from an indwelling cannula in each subject after 12-h fasting. All blood samples collected were centrifuged at the CRU and sera were stored at −80 °C until transported to a certified institutional laboratory. The glucose homeostasis parameters (fasting plasma glucose, glycated hemoglobin (HbA1c), C-peptide, and insulin), lipid profile (total cholesterol, high-density lipoprotein cholesterol, low-density lipoprotein cholesterol, and triglycerides), and other routine laboratory parameters (urea, creatinine, albumin, CRP, lactate, cortisol, TSH, fT4, anti-TPO, blood count, D-dimers) were evaluated in a certified hospital laboratory. Fasting plasma glucose was assessed using the hexokinase reaction (KONELAB, Dreieich, Germany); C-peptide by using a competitive solid-phase chemiluminescent enzyme immunoassay (Immulite 2000, Los Angeles, CA, USA); HbA1c by using high-pressure liquid boronate affinity chromatography (Primus Corporation, Kansas City, MO, USA); insulin by using a competitive solid-phase chemiluminescent enzyme immunoassay (Immulite 2000, Los Angeles, CA, USA); total cholesterol and triglycerides using an enzymatic method kit (KONELAB, Dreieich, Germany); high-density lipoprotein-cholesterol (HDL-c) measured using a polyethylene glycol-modified enzymatic assay kit (ROCHE, Basel, Switzerland); and low-density lipoprotein–cholesterol (LDL-c) calculated using the standard Friedewald equation. The beta cell-specific autoAb (antiIA2, GADA) were analyzed using ELISA (Medipan GmbH, Germany). Serum NEFA and glycerol were measured using an enzyme colorimetric kit (Randox Laboratories Ltd., UK) and serum branched-chain amino acids (BCAA) were measured using countercurrent ELFO [25].

#### Insulin sensitivity, secretion indices, and hyperglycemia

An oral glucose tolerance test (OGTT, 75 g glucose) was performed after fasting overnight (12 h) fasting and following standard WHO recommendations. First, baseline blood samples were obtained from an indwelling cannula, then 15, 30, 60, 90, and 120 min after ingestion producing 6 values for each subject. Insulin sensitivity and secretion were evaluated using baseline values of serum glucose, insulin, and C peptide (HOMA indices [26]) and data from the oral glucose tolerance test (OGTT). Incremental AUCs for glucose and insulin were calculated using the trapezoid rule. Insulin sensitivity alone was expressed as HOMA indices and Matsuda insulin sensitivity index (ISI) as published [27]. Insulin secretion was expressed as the insulinogenic index (IGI). IGI was calculated using the change in insulinemia over glycemia in 0 to 30 min: ΔINS 30-0/ΔGLU 30-0 (μU/mL*mg/dL). To specifically assess beta cell function, the oral disposition index (DI) was calculated to adjust for actual insulin sensitivity as IGI*ISI in each participant [28].

Hyperglycemia was defined as fasting glycemia ≥5.6 mM and/or 2 h OGTT glycemia ≥7.8 mM. Diabetes was defined as fasting glycemia ≥7.6 mM and/or 2 h OGTT glycemia ≥11.1 mM [29].

#### Resting energy expenditure and substrate preference

Indirect calorimetry was measured in each subject: (1) after fasting overnight (12 h) fasting and 30 min of bed rest and (2) in 100–120 min of OGTT, using a canopy ventilated hood system (QuarkRMR, Cosmed, Italy). Measurements were made for 20 min after stabilization of the initial ventilation, data were averaged per 30 s, and a percent variance was recorded to confirm that the subjects were in a steady state. Gas sensors were calibrated using a mixture of known concentrations of gases and ambient air, and the flowmeter was calibrated using a semiautomated pump. All calibrations were done before each respective measurement. The measured ambient air temperature, pressure, and humidity were recorded. All subjects were asked to collect urine for 24 h before measurement, and the concentration of urea in the mixed sample was used to calculate the nitrogen output. Measured VO2 and VCO2 and fat-free mass were used to calculate daily non-protein resting energy expenditure using the Weir formula (REE) and respiratory quotient (RQ). VO2, VCO2, and nitrogen loss per 24 h were used to calculate basal substrate utilization of carbohydrates, fat, and protein [30, 31]. The Harris-Benedict equation with fat-free mass weight was used to estimate predicted REE [32]. The change in RQ from baseline to 120 min OGTT (ΔRQ 120-0) was calculated as a parameter of metabolic flexibility and the change in REE (ΔREE 120-0) as a parameter of diet-induced thermogenesis.

#### Statistics

Normally distributed data are presented as mean and standard deviation (SD), skewed distributed data as the median and interquartile range (IQR), and categorical variables as numbers and percentages. The mean differences with 95% confidence intervals (CI) between the normal glycemia and hyperglycemia groups were calculated using unpaired T-tests for normally distributed variables and the median differences with 95% CI using the Wilcoxon signed-rank test for skewed distributed variables. The differences between the groups were adjusted for age, sex, and BMI at T0 or T6, respectively.

The time differences between T6 and T0 were investigated with the paired T-test for continuous variables, and the time differences between the three visits and between groups were determined using generalized linear models (repeated measures ANOVA).

To investigate whether the exclusion of the extreme outliers from our main analysis could have influenced our results, we performed a sensitivity analysis by including the entire study sample (*n* = 37 at T0, *n* = 31 at T6; compare: Figure S1). Therefore, all analyses were repeated on the entire study sample. Differences between groups or between baseline and follow-up visits were determined according to the precision of the 95% CI (null value not included) and the corresponding *p*-value (*p* < 0.05). All analyses were performed in SAS (version 9.4; SAS Institute, Cary, USA).

## Results

### Clinical characteristics

Out of 37 patients, survivors of severe COVID-19, 26 patients with complete outcome data were included in the main analysis (Figure S1). The patients were examined on average 21 ± 6.5 days after COVID-19 diagnosis. Patients had a respiratory failure that required high flow (*n* = 19) or low flow (*n* = 18) nasal oxygen therapy. High-dose corticosteroids were administered to the patients in most cases (dexamethasone: *n* = 23; prednisone: *n* = 9; methyl-prednisolone: *n* = 1; no corticosteroids: *n* = 4) during hospitalization. All patients were without any corticosteroid treatment at 3 and 6 months of the follow-up. The clinical characteristics are summarized in Table 1 for the analysis sample (*n* = 26) and in Supplement Table 1 for the total population without excluding patients with missing data.

**Table 1:** Clinical characteristics of the study population (*n* = 26).

	T0 aseline	T3 Visit after 3 months	T6 Visit after 6 months	*p*
n	26	26	26	–
Age [years]	59.0 ± 9.9	59.0 ± 9.9	59.0 ± 9.9	–
Sex [*n* female, %]	9 (35%)	9 (35%)	9 (35%)	–
Prevalence hyperglycemia [*n*, %]	**17 (65%)**	n.a.	**13 (50%)**	0.04
BMI [kg/m²]	**30.4** **±** **4.8**	**31.6** **±** **4.7**	**31.6** **±** **4.6**	<0.0001
Body weight [kg]	**91.0** **±** **15.4**	**94.3** **±** **15.9**	**94.7** **±** **15.3**	<0.0001
WHR	0.94 ± 0.08	0.94 ± 0.08	0.93 ± 0.08	0.11
Fat mass [%]	24 ± 10	24 ± 10	26 ± 13	0.30
Systolic blood pressure [mmHg]	132 ± 16	133 ± 16	135 ± 18	0.37
Diastolic blood pressure [mmHg]	84 ± 7	85 ± 8	84 ± 9	0.98
First random glucose [mmol/L]	6.5 (6.0, 7.5)	–	–	–
Fasting glucose [mmol/L]	5.4 ± 1.1	5.7 ± 0.5	5.4 ± 0.6	0.83
Glucose after 2 h [mmol/L]	**9.1** **±** **2.5**	n.a.	**7.6** **±** **1.8**	<0.001
Fasting insulin [mIU/L]	**13.0 (9.1, 17.5)**	**14.2 (10.9, 16.7)**	**7.7 (6.2, 13.4)**	0.01
Insulin after 2 h [mIU/L]	77.8 (49.5, 109.2)	n.a.	48.2 (36.7, 121.9)	0.06
Fasting C-peptide [pmol/L]	**890** **±** **272**	**706** **±** **265**	**558** **±** **215**	<0.0001
C-peptide after 2 h [pmol/L]	**3340** **±** **792**	n.a.	**2312** **±** **716**	<0.0001
Fasting NEFA [mmol/L]	**0.96** **±** **0.25**	n.a.	**0.77** **±** **0.27**	0.001
NEFA after 2 h [mmol/L]	**0.57** **±** **0.20**	n.a.	**0.34** **±** **0.14**	<0.0001
Fasting glycerol [µmol/L]	**260** **±** **72**	n.a.	**156** **±** **47**	<0.0001
Glycerol after 2 h [µmol/L]	**218** **±** **53**	n.a.	**121** **±** **25**	<0.0001
Insulinogenic index	1.17 (0.85, 1.70)	n.a.	1.49 (0.81, 1.53)	0.29
Insulin sensitivity index	**3.23** **±** **1.41**	n.a.	**4.53** **±** **2.30**	0.003
Disposition index	4.83 ± 4.20	n.a.	4.89 ± 2.58	0.86
HOMA-beta	**1.59 (1.14, 2.50)**	n.a.	**1.01 (0.81, 1.53)**	0.01
HOMA-IR	**2.62 (1.97, 4.93)**	n.a.	**1.77 (1.48, 3.42)**	0.01
HbA1c [mmol/mol]	**47.4** **±** **13.4**	35.6 ± 4.3	**37.8** **±** **4.2**	<0.001
Triglycerides [mmol/L]	1.76 ± 0.89	1.72 ± 1.28	1.50 ± 0.83	0.08
Total cholesterol [mmol/L]	4.36 ± 0.84	5.19 ± 1.14	4.77 ± 0.92	0.11
HDL cholesterol [mmol/L]	1.30 ± 0.34	1.20 ± 0.26	1.19 ± 0.33	0.16
LDL cholesterol [mmol/L]	**2.26** **±** **0.77**	**3.31** **±** **1.06**	**2.91** **±** **0.90**	0.01
ALT [μkat/L]	**1.11** **±** **0.58**	**0.49** **±** **0.18**	**0.46** **±** **0.19**	<0.0001
AST [μkat/L]	**0.49** **±** **0.20**	**0.38** **±** **0.09**	0.36 ± 0.11	0.001
Urea [mmol/L]	5.59 ± 2.80	5.40 ± 1.47	5.76 ± 1.42	0.73
Creatinine [μmol/L]	**68.7** **±** **15.4**	**74.4** **±** **15.1**	**75.8** **±** **15.6**	<0.001
Albumine [g/L]	**40.3** **±** **1.9**	**47.1** **±** **6.1**	**43.0** **±** **2.6**	<0.0001
Cortisol [nmol/L]	258 ± 184	n.a.	249 ± 99	0.81
TSH [µmol/L]	**1.89 (1.50, 2.66)**	n.a.	**1.51 (1.23, 1.85)**	<0.001
Triiodothyronine (T3) [pmol/L]	**5.25** **±** **0.72**	n.a.	**5.74** **±** **0.52**	0.01
Thyroxine (T4) [pmol/L]	**15.9** **±** **3.7**	n.a.	**14.5** **±** **1.9**	0.03
White blood cells [×10^9^/L]	**9.65** **±** **4.11**	**6.68** **±** **1.65**	**6.08** **±** **1.67**	<0.001
Platelets [×10^9^/L]	234 ± 112	247 ± 61	211 ± 57	0.26
D-dimer [μg/L]	**925 (340, 2140)**	**590 (310, 860)**	**250 (210, 530)**	<0.001
Baecke Score	**6.54** **±** **1.31**	**7.61** **±** **1.56**	**7.58** **±** **1.49**	0.005

Normally distributed variables were expressed as mean ± standard deviation and skewed distributed variables as median and interquartile range.

*P*-values for differences between T0 and T6 were calculated with paired T-test for normal distributed variables, with Wilcoxon signed-rank test for skewed distributed variables and via Chi-square test for categorical variables.

Statistically significant values are shown in bold.

The presence and persistence of symptoms related to COVID-19 are depicted in Table S6. Almost all patients reported the presence of some post-COVID-19-related symptom, the most common being fatigue and muscle weakness. At T6, about one-third of the patients had at least one remaining symptom (mainly fatigue, joint pain, and hair loss). The prevalence of hyperglycemia at baseline was 65% (17 patients). Seven patients had baseline HbA1c above 48 mmol/mol (all in the hyperglycemic group in the main analysis). Hyperglycemia remained as high as 50% (13 patients) after 6 months. Of these patients, 10 were classified as having prediabetes (treated with lifestyle intervention only) and 3 had been diagnosed with Type 2 diabetes and were on pharmacological treatment (metformin *n* = 2, metformin, and gliflozin *n* = 1). Of note these patients all had HbA1c above 48 mmol/mol.

We found no patient with a positive antibody titer, neither in GADA (>5.5 ± 0.5 IU/L) nor in antiIA2 (>3.7 ± 0.4 IU/L).

### Insulin function measures

To gain insight into glycemic outcomes, we compared patients with normal glycemia vs. hyperglycemia at T0 and T6. The clinical characteristics of the groups are summarized in Table S2. The groups were comparable in a wide range of parameters, however, they differed in fasting and postprandial glycemia, and HbA1c.

**Fig. 1:**
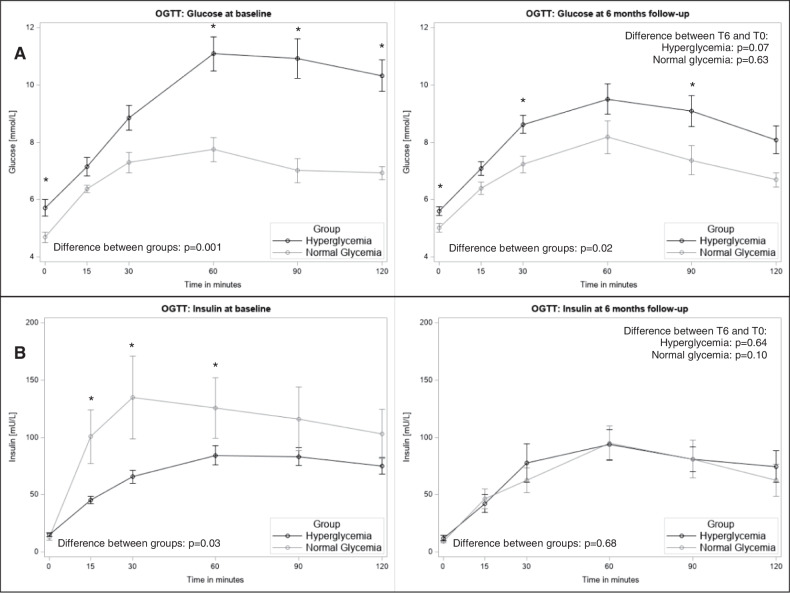
Oral glucose tolerance test (OGTT) curves for glucose and insulin in the analysis population with complete outcome data (*n* = 26). Group differences between the normal glycaemic (gray) and hyperglycaemic (black) groups were tested with unpaired T-test for the single time points of the OGTT curves and differences between T6 and T0 with repeated measures ANOVA. Data are shown for **A** glucose and **B** insulin at baseline and at 6 months follow-up, respectively, *p < 0.05 indicating group differences at single time points. OGTT, oral glucose tolerance test; T0, baseline visit; T6, visit after six months.

A worse glycemic response (Fig. 1A) combined with a lower insulinogenic response, dominantly in the first 30 min (Fig. 1B) was observed in the hyperglycemia group. Although the insulinogenic response declined and was comparable between the groups at T6 (Fig. 1B), the glycemic response remained worse for the hyperglycemic group (Fig. 1A). This translated to the calculated glycemic indices as summarized in Table 2. The hyperglycemic group had worse insulinogenic and disposition indices at baseline (IGI_H_ = 1.01 ± 0.51, IGI_N_ = 2.78 ± 1.77, *p* = 0.004; DI_H_ = 3.05 ± 1.79 vs DI_N_ = 8.40 ± 5.42, *p* = 0.003), the difference that was not apparent at T6. Differences in insulin sensitivity changes (both ISI Matsuda and HOMA indices) improved only in the hyperglycemic group [ΔT6-T0 mean difference for ISI (95% CI): 1.84 (0.45, 3.24), *p* = 0.01] (Table 2).

**Table 2:** Outcome indices at baseline (T0) and follow-up visit after 6 months (T6) and the mean differences plus 95% CI between these visits in the normal glycemic and hyperglycemic groups of the study population.

	Normal glycemia	Hyperglycemia	Adjusted group differences	
	Mean ± SD	Mean ± SD	Mean difference (95% CI)	*p*
	**T0 (*n*** = **26)**			
Insulinogenic index	2.78 ± 1.77	1.01 ± 0.51	**−1.70 (−2.78, −0.63)**	0.004
Insulin sensitivity index	3.47 ± 1.78	3.12 ± 1.23	−0.52 (−1.91, 0.86)	0.44
Disposition Index	8.40 ± 5.42	3.05 ± 1.79	**−5.64 (−9.11, −2.16)**	0.003
HOMA-beta	1.63 ± 1.07	2.00 ± 0.92	0.61 (−0.26, 1.47)	0.16
HOMA-IR	2.69 ± 1.87	4.03 ± 2.44	1.84 (−0.15, 3.84)	0.07
	**T6 (*n*** = **26)**			
Insulinogenic index	1.35 ± 0.64	1.57 ± 2.28	0.52 (−1.32, 2.36)	0.56
Insulin sensitivity index	4.56 ± 1.27	4.52 ± 2.75	−0.62 (−2.67, 1.43)	0.53
Disposition Index	5.67 ± 1.98	4.45 ± 2.83	−1.11 (−3.47, 1.25)	0.34
HOMA-beta	1.20 ± 0.41	1.60 ± 1.24	0.62 (−0.35, 1.58)	0.20
HOMA-IR	2.04 ± 0.62	3.20 ± 2.83	1.59 (−0.59, 3.78)	0.14

	ΔT6-T0 (*n* = 26)			
	Normal glycemia		Hyperglycemia	
	Mean difference (95% CI)	*p*	Mean difference (95% CI)	*p*
Insulinogenic index	**−1.34 (−2.48, −0.19)**	0.03	0.54 (−0.72, 1.79)	0.38
Insulin sensitivity index	1.02 (−0.17, 2.22)	0.08	**1.84 (0.45, 3.24)**	0.01
Disposition index	−2.39 (−6.73, 1.96)	0.23	1.05 (−0.33, 2.44)	0.12
HOMA-beta	−0.43 (−1.12, 0.27)	0.20	**−0.48 (−0.90, −0.05)**	0.03
HOMA-IR	−0.65 (−1.88, 0.59)	0.26	**−0.99 (−1.90, −0.09)**	0.03

Data are shown as mean ± SD. Differences between the normal glycaemic and hyperglycaemic group are shown as mean difference ± 95% CI adjusted for age, sex, and BMI. *P*-values were derived from unpaired T-test.

Differences over time between T6 and T0 were derived from paired T-test.

*CI* confidence interval, *HOMA-beta* homeostasis model assessment of beta cell function, *HOMA-IR* homeostasis model assessment of insulin resistance, *SD* standard deviation, *T0* baseline visit, *T6* visit after six months, *ΔT6-T0* change between T6 and T0.

Bold values denote statistically significant *p* values.

**Fig. 2:**
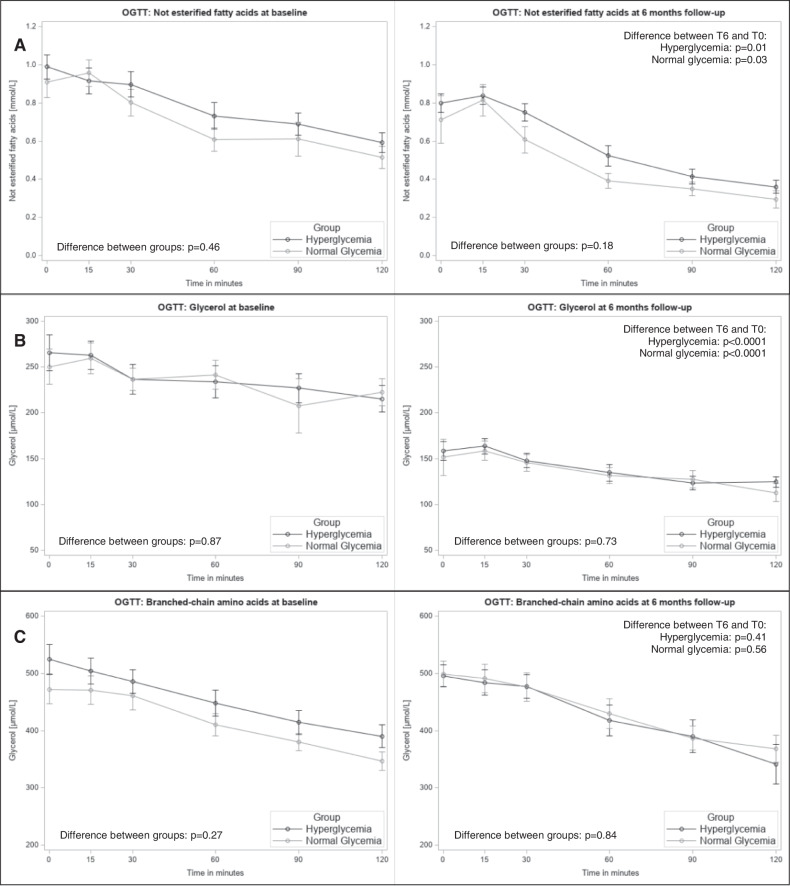
Oral glucose tolerance test (OGTT) curves for non-esterified fatty acids, glycerol, and branched-chain amino acids in the analysis population with complete outcome data (*n* = 26). Group differences between the normal glycaemic (gray) and hyperglycaemic (black) groups were tested with unpaired T-test for the single time points of the OGTT curves and differences between T6 and T0 with repeated measures ANOVA. Data are shown for **A** non-esterified fatty acids, **B** glycerol, and **C** branched-chain amino acids at baseline and at 6 months follow-up, respectively. *p < 0.05 indicating group differences at single time points. OGTT, oral glucose tolerance test; T0, baseline visit; T6, visit after six months.

In the same settings, we analyzed circulating NEFA and glycerol as whole-body indices of basal and insulin-stimulated lipolysis (Fig. 2A, B). Both the basal values and the kinetics of the response to OGTT were comparable between the groups at both T0 and T6. On the other hand, both groups had comparable lower basal and stimulated values at T6.

Neither the basal values of BCAA nor the suppression of its levels in OGTT showed any difference between the groups and T0-T6 (Fig. 2C).

### Weight balance, resting energy expenditure, and substrate oxidation

The patients were examined at baseline in the weight loss phase. The weight loss in acute disease was 6.1 ± 4 kg (~7% of the pre-hospitalization weight) which was associated with a reduction in self-reported oral intake of 86 ± 31% (*n* = 26). Total body weight increased over 6 months by 3.6 ± 3.2 kg (*p* < 0.0001, *n* = 26).

**Fig. 3:**
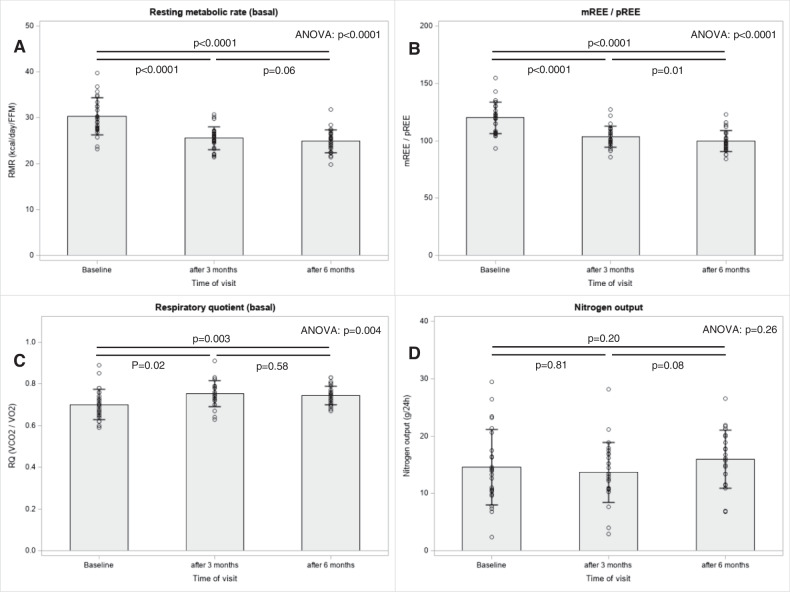
Bar charts for resting energy expenditure parameters of the indirect calorimetry in the analysis population with complete outcome data (*n* = 26). Differences in **A** basal resting metabolic rate, **B** metabolic to the predicted rate of resting energy expenditure, **C** basal respiratory quotient, and **D** nitrogen output over time were tested with repeated measures ANOVA and are shown as *p*-values. FFM, fat-free mass; mREE, measured resting energy expenditure; pREE, predicted resting energy expenditure; RMR, resting metabolic rate; RQ, respiratory quotient; VCO2, carbon dioxide production; VO2, oxygen consumption.

**Fig. 4:**
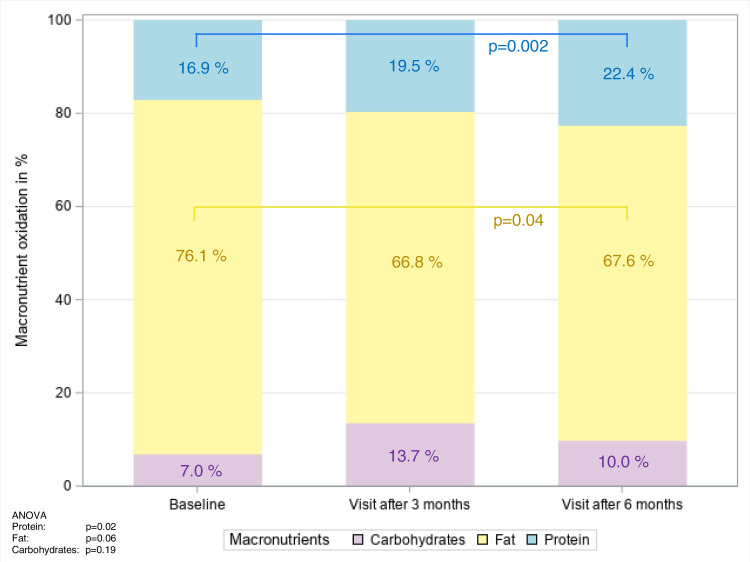
Oxidation of carbohydrates, fat, and protein expressed as a share from 100% at baseline and follow-up after 3 months and 6 months in the analysis population with complete outcome data (*n* = 26). Differences between macronutrient oxidation at baseline, visit after 3 months, and after 6 months were tested with repeated measures ANOVA, *p-values < 0.05 indicating differences in time for macronutrient oxidation between two-time points for fat between baseline and visit after 6 months (*p* = 0.04), as well as for protein between baseline and visit after 6 months (*p* = 0.002).

For REE analyses, the whole group was compared in terms of measured over-predicted REE per kg of fat-free mass at baseline, 3 and 6 months of follow-up. The data are summarized in Table 3 and Figs. 3 and 4. At baseline, REE was 30.3 ± 4.0 kcal per kg of active tissue mass, which was ~19% above the predicted values from the Harris-Benedict equation; REE decreased to the predicted values over 3 months (Fig. 3). The mean basal RQ was 0.70 ± 0.07 at baseline and slightly increased to 0.74 ± 0.05 (*p* = 0.003) at 6 months (Table 3). We did not observe differences over time in either ΔRQ_120-0_ or ΔREE_120-0_ (Table 3). The oxidation of the individual substrates is shown in Fig. 4. The predominant substrate oxidized under fasting conditions was fat. However, fat oxidation decreased slightly from T0 to T6 from ~76% to ~68% (*p* = 0.04), whereas protein oxidation increased from ~17% to ~22% over 6 months (ANOVA: *p* = 0.02).

**Table 3:** Clinical metabolic variables at baseline (T0) and follow-up visit after 3 (T3) and 6 months (T6) and the mean difference plus 95% CI between T0 and T6 in the study population.

	T0		T3		T6		ΔT6-T0		
	*n*	Mean ± SD	*n*	Mean ± SD	*n*	Mean ± SD	*n*	Mean difference (95% CI)	*p*
REE_Harris Benedict_ [kcal/d]	26	1718 ± 270	25	1760 ± 288	26	1767 ± 276	26	**49 (31, 67)**	<0.0001
REE_0_ [kcal/d]	26	2052 ± 329	25	1819 ± 292	26	1759 ± 285	26	**−293 (−378, −207)**	<0.0001
REE_120_ [kcal/d]	25	2148 ± 313		n.a.		1924 ± 277	25	**−236 (−338, −134)**	<0.0001
ΔREE_120-0_ [kcal/d]	25	110 ± 188		n.a.	25	165 ± 116	25	54 (−30, 139)	0.20
REE_0_/REE_Harris Benedict_ [%]	26	120 ± 14	25	104 ± 9	26	100 ± 9	26	**−20 (−25, −15)**	<0.0001
REE_0_/ATM ratio [kcal/kg]	26	30.3 ± 4.0	25	25.5 ± 2.5	26	24.8 ± 2.6	26	**−5.4 (−6.8, −4.1)**	<0.0001
RQ_0_	26	0.70 ± 0.07	25	0.75 ± 0.06	26	0.74 ± 0.05	26	**0.04 (0.02, 0.07)**	0.003
RQ_120_	25	0.80 ± 0.09		n.a.	25	0.82 ± 0.05	25	0.02 (−0.02, 0.06)	0.35
ΔRQ_120-0_	25	0.09 ± 0.08		n.a.	25	0.07 ± 0.05	25	−0.03 (−0.06, 0.01)	0.12

Data are shown as mean ± SD. Differences between T6 and T0 are shown as mean difference ± 95% CI. *P*-values were derived from paired T-test.

*ATM* active tissue mass, *CI* confidence interval, *n.a.* not applicable, *REE* resting energy expenditure, *RQ* respiratory quotient, *SD* standard deviation, *T0* baseline visit, *T3* visit after three months, *T6* visit after six months.

Bold values denote statistically significant *p* values.

### Sensitivity analyses

Sensitivity analyses were conducted on the entire study population including the five participants with extreme outliers (*n* = 37). There were no noticeable differences in characteristics when comparing the total study population with the study population excluding extreme outliers (Table S1). The findings on the clinical variables (Table S3) and energy expenditure (Table S4) were also comparable. When comparing the findings on insulin secretion indices, only slight differences were observed between the main and sensitivity analysis. Although in the main analysis, a clear reduction in IGI was observed from baseline to six months of follow-up [−1.34 (95% CI −2.48, −0.19); *p* = 0.03] in the normal glycemic group (Table 2), the reduction was not accurately estimated in the sensitivity analysis [−0.85 (95% CI −2.59, 4.75); *p* = 0.41] (Table S5).

## Discussion

The main findings of the current study are that (1) highly prevalent hyperglycemia is caused by an insufficient increase in insulin secretion to compensate for the prevailing IR, (2) hyperglycemia persists as classified as prediabetes or diabetes in a large proportion of patients despite improvement in their IS, and (3) REE is high during the acute phase and reverts to predicted levels already after 3 months of follow-up.

### Hypermetabolism is present in the acute phase but does not persist over time

We observed an approximately 20% higher than predicted energy expenditure across the entire sample in the acute phase. These findings are in line with the results of other studies showing hypermetabolism in the acute phase of COVID-19 phase [33–36] For example, Yu et al. found that COVID-19 patients have energy expenditure as high as 200% of predicted [36]. This increased energy expenditure in patients with COVID-19 was even higher than the energy expenditure observed in patients with burn wounds or acute respiratory distress syndrome, which are both conditions associated with high REE and increased nutritional needs [37]. Hypermetabolism was suggested to persist in post-COVID-19 patients [38]. In contrast, we found that already after 3 months of follow-up, the REE returned to the predicted values. Unexpectedly, we observed a slightly increased level of protein oxidation after 6 months. As we do not have data from the diet record, we cannot conclude whether this reflects endogenous protein catabolism or anabolic resistance to relatively high protein refeeding in these patients.

Mechanisms of increased REE in patients with severe COVID-19 are not well understood, but in general, activation of immune cells, medication, and increased body temperature can increase energy expenditure in critically ill patients [39]. High-dose corticosteroids are the main treatment for COVID-19 pneumonia, and acute administration of cortisol has been shown to increase energy expenditure [40]. It is noteworthy that REE measurements in oxygen-dependent patients with highly transmissible diseases are challenging and make it difficult to obtain comparable prospective data because breath-by-breath measurements during artificial ventilation may yield different results than when the canopy is used. We were able to measure REE using a ventilated canopy system as soon as the patients were weaned from the oxygen supply, so we did not capture the most critical phase of the disease; moreover, we focused only on survivors, which may explain the relatively smaller difference between measured and predicted REE than in previous reports.

### Hyperglycemia is a common feature in acute severe COVID-19

The prevalence of hyperglycemia in the current study was as high as 63% (*n* = 20) in a group of patients without previously diagnosed diabetes. In a meta-analysis, the prevalence of hyperglycemia in acute COVID-19 was 14.4% in the population of unselected patients [41] and as high as 85% in patients with critical illness [10]. However, it is difficult to conclude from these epidemiological data whether newly diagnosed diabetes is truly new-onset diabetes or a manifestation of diabetes that has not been diagnosed in the past. There were 7 patients in the hyperglycemic group (the main analysis) with HbA1c levels 48 mmol/mol at baseline, and we cannot rule out that these patients have had diabetes for a long time that had not been diagnosed in the past. Moreover, it has been shown that other factors the presence of diabetes could influence HbA1c levels [42], interestingly, COVID-19 itself could be associated with falsely positive HbA1c [43] We did not find a single patient with islet autoantibodies, which makes it unlikely that we enrolled a patient with new-onset type 1 diabetes in the study.

### Hyperglycemia is related both to insulin resistance and beta cell failure

We compared two groups of patients with normal glycemia and hyperglycemia to explore the contribution of insulin resistance and beta cell failure to the manifestation of hyperglycemia. The main difference we observed in the acute phase of COVID-19 was relatively lower insulin secretion 30 and 60 min after glucose loading, suggesting a primary defect in the first phase of the insulin response. This also resulted in lower insulinogenic and disposition indices.

These findings are in contradiction with the current literature [9, 10]. The findings of Reiterer et al. [10] showed that insulin resistance is the main cause of hyperglycemia in critically ill patients with COVID-19. Here, the discrepancies could be explained by the fact that in the study only the ratio of C-peptide to glucose levels was used to classify the differences between insulin sensitivity and secretion, but the dynamics of insulin secretion under stimulated conditions were not measured. Moreover, the authors admit that they had to rely on nonfasted samples, which cannot preclude that the values may reflect an insulin-stimulated state. Another study from Montefusco et al. [9] pointed toward maintaining insulin secretion when evaluated by an arginine stimulation test. Here, the discrepancy could be caused by different source populations (severe vs. non-severe COVID-19) and different designs, as we compared hyperglycemic vs. normoglycemic subjects, while the study [9] patients with acute and post-acute COVID-19 that were examined were not stratified. Actually, in their sample of 10 vs. 10 subjects, there is a wide distribution of the response.

Concerning other potential mechanisms, we investigated corticosteroid treatment during acute COVID-19. High-dose corticosteroids were used in all but four patients in the hyperglycemia group. The effects of administered corticosteroids were measured by endogenous cortisol suppression that was comparable between groups. Corticosteroids were shown to affect IR [44], even in a dose-response manner, and administration was shown to precipitate diabetes development [45]. On the other hand, short-term corticosteroid treatment probably does not influence insulin secretion [46] which determined hyperglycemia in the current study. Therefore, we conclude that corticosteroids alone would not explain the incident hyperglycemia, but they may have contributed to an overall decline in IS at T0. Of note, persistent suppression of endogenous cortisol was still present after 6 months, and therefore it is likely that some of the post-covid symptoms overlap with adrenal insufficiency.

### Insulin resistance of adipose tissue does not contribute to systemic IR

Above, we focused on glycemic regulation that mainly reflects IR and insulin response of the liver and skeletal muscle, but adipose tissue has also been shown to be an important organ that contributes to systemic IR in patients with COVID-19 [10]. Among putative mechanisms, SARS-CoV-2 virus replication in adipocytes, leading to AT inflammation, and lower adiponectin and adiponectin/leptin ratios were suggested [8, 10]. To address the contribution of AT, we compared the suppression of circulating NEFA and glycerol during OGTT. We found that both NEFA and glycerol suppressibility increased at T0 versus T6 at all time points in OGTT but similarly in both groups. In conclusion, indices of increased basal lipolysis do not relate to the presence/absence of hyperglycemia. We also analyzed changes in BCAA levels. BCAA were shown to be among important precursors for de novo lipogenesis and their circulating levels may reflect resistance to insulin function on protein turnover [47]. We found no differences between groups and no change during the 6-month follow-up, indicating that protein turnover could have remained unchanged.

### Hyperglycemia persists throughout follow-up and there is a high prevalence of pre-/diabetics in the post-COVID phase

After 6 months, insulin sensitivity improved in both groups, but more markedly in the hyperglycemic group, while beta cell function (measured as disposition index) remained unchanged. Therefore, at 6 months of follow-up, 13 hyperglycemic patients remained in the group (50%). Three of these patients were classified as having persistent Type 2 diabetes and pharmacological treatment was prescribed, and 10 patients were classified as having prediabetes and received lifestyle intervention only.

### Strengths and limitations

Despite the strengths of the current study, namely prospective 6-month follow-up and detailed metabolic phenotyping in survivors of severe COVID-19, other important limitations need to be discussed. We have encountered an important dropout to follow-up (*n* = 6; 18.8%) that limited statistical analysis, such as stratified analysis by sex or age groups. We included only survivors of COVID-19, within 4 weeks after COVID-19 diagnosis, so we may have missed the period of critical illness when signs of deterioration of insulin function and hypermetabolism may be more pronounced and prevalent. This limitation stemmed from feasibility reasons, as performing OGTT and indirect calorimetry in unstable patients is challenging, and we also wanted to eliminate the mortality dropout rate at baseline. The design of the study, i.e., without an adequate comparator group (like other severely ill for T0 and healthy for T6), makes it difficult to infer conclusions on causality between COVID-19 and hyperglycemia and is prone to residual confounding. During the recruitment period, there were only a few critically ill patients without COVID-19 and therefore the comparator was not feasible. As mentioned above, we cannot conclude the separation between new-onset and newly diagnosed diabetes. Besides the disease itself, there are further factors that could be associated with a change in glucose tolerance, i.e., physical activity and dietary intake. Physical activity increased from baseline to the follow-up, though the increase is not clinically meaningful, it may have contributed to the improvement of IR. We cannot conclude about the eventual change in eating habits as we did not collect data on dietary intake over the follow-up period. And lastly, the 6-month follow-up period is relatively short to see further clinical evolution of pre/diabetes.

## Conclusion

To conclude, hyperglycemia is prevalent in patients with severe COVID-19 and relates to inadequate beta cell response to match insulin resistance in acute disease. Improvement in insulin resistance leads to improvement in glucose tolerance over six months. Hypermetabolism is probably associated with the critical state of severe COVID-19 and its treatment with corticosteroids and normalizes relatively quickly during recovery. Given the findings, patients after severe COVID-19 should be followed-up and actively screened for diabetes in the post-COVID phase. Corticosteroids should be given for the shortest period possible, and patients should be checked for potential clinical signs of adrenal suppression. Despite all the uncertainties that remain regarding COVID-19 and diabetes, it is clear that these two pandemics are intertwined and closely interact. Diabetes is one of the main risk factors for severe COVID-19 disease, and, on the other hand, COVID-19 may induce diabetes in patients with impaired beta cell function. Therefore, further research into mechanisms of beta cell dysfunction during COVID-19 is warranted.

## Supplementary information

Supplemental Material

## Data Availability

Pseudonymized datasets generated and analyzed during the current study are available from the corresponding author upon reasonable request.
