# Supplementary material for: Severe COVID-19 associated hyperglycemia is caused by beta cell dysfunction: a prospective cohort study
Source: Nutr Diabetes. 2023 Jul 17;13:11. doi: 10.1038/s41387-023-00241-7 (PMC10352285; doi:10.1038/s41387-023-00241-7)
Supplement: Supplementary file 1 — Supplemental Material [file 41387_2023_241_MOESM1_ESM.pdf]

Severe COVID-19 associated hyperglycemia is caused by beta cell dysfunction: a prospective cohort study

Jan Gojda[1]*, Kateřina Koudelková[1], Anna Ouřadová[1], Alexander Lang[2,3], Magdaléna Krbcová[1], Alexandra Gvozdeva[1], Viktor Šebo[4], Lotte Slagmolen[5], Jana Potočková[1], Petr Tůma[6], Lenka Rossmeislová[2], Michal Anděl[1], Fredrik Karpe[7], Sabrina Schlesinger[3,4]

1/ Dept. of Internal Medicine, Third Faculty of Medicine, Charles University, and Královské Vinohrady University Hospital, Prague, Czech Rep.

2/ Institute for Biometrics and Epidemiology, German Diabetes Center (Deutsches Diabetes-Zentrum/DDZ), Leibniz Center for Diabetes Research at Heinrich Heine University Düsseldorf, Düsseldorf, Germany.

3/ German Center for Diabetes Research (DZD), Partner Düsseldorf, Düsseldorf, Germany.

4/ Dept. of Pathophysiology, Third Faculty of Medicine, Charles University, Prague, Czech Rep.

5/ Faculty of Movement and Rehabilitation Sciences, Katholieke Universiteit Leuven, Belgium

6/ Department of Hygiene, Third Faculty of Medicine, Charles University, Prague, Czech Rep.

7/ Oxford Center for Diabetes, Endocrinology, and Metabolism, University of Oxford, United Kingdom

*Corresponding author: Jan Gojda, MD, Ph.D., Department of Internal Medicine, Kralovske Vinohrady University Hospital and Third Faculty of Medicine, Charles University, Prague, Czech Republic. e-mail: jan.gojda@lf3.cuni.cz

Keywords

Sars-CoV-2, hyperglycemia, diabetes, insulin resistance, beta cell dysfunction, hypermetabolism

Supplement Table 1: Sensitivity Analysis: Clinical characteristics of the full data-set population (n=37)

	T0<br>Baseline	T3<br>Visit after 3 months	T6<br>Visit after 6 months	p
n	37	32	31	-
Age [years]	56.3 ± 10.3	56.8 ± 10.6	57.7 ± 9.9	-
Sex [n female, %]	14 (38%)	13 (41%)	11 (36%)	-
Prevalence hyperglycemia [n, %]	22 (59%)	n.a.	14 (45%)	0.06
BMI [kg/m²]	30.5 ± 5.1	31.6 ± 4.9	31.4 ± 4.4	<0.0001
Body weight [kg]	90.6 ± 16.4	93.9 ± 14.7	94.9 ± 14.1	<0.0001
WHR	0.94 ± 0.08	0.92 ± 0.08	0.92 ± 0.08	0.05
Fat mass [%]	25 ± 10	24 ± 10	26 ± 12	0.36
Systolic blood pressure [mmHg]	131 ± 15	131 ± 16	134 ± 17	0.59
Diastolic blood pressure [mmHg]	84 ± 7	84 ± 9	84 ± 9	0.93
First random glucose [mmol/L]	6.3 (5.9, 7.2)	-	-	-
Fasting glucose [mmol/L]	5.3 ± 1.1	5.7 ± 0.5	5.4 ± 0.6	0.66
Glucose after 2 hours [mmol/L]	8.5 ± 2.6	n.a.	7.4 ± 1.7	<0.001
Fasting insulin [mIU/L]	11.3 (9.1, 15.8)	14.6 (11.4, 16.9)	7.7 (6.2, 13.4)	0.003
Insulin after 2 hours [mIU/L]	85.6 (49.5, 109.2)	n.a.	41.9 (24.6, 113.5)	0.02
Fasting C-peptide [pmol/L]	910 ± 336	697 ± 252	543 ± 206	<0.0001
C-peptide after 2 hours [pmol/L]	3386 ± 1008	n.a.	2152 ± 759	<0.0001
Fasting NEFA [mmol/L]	0.93 ± 0.25	n.a.	0.74 ± 0.26	0.001
NEFA after 2 hours [mmol/L]	0.55 ± 0.20	n.a.	0.32 ± 0.14	<0.0001
Fasting glycerol [µmol/L]	251 ± 71	n.a.	153 ± 45	<0.0001
Glycerol after 2 hours [µmol/L]	215 ± 62	n.a.	118 ± 25	<0.0001
Insulinogenic index	1.32 (0.85, 2.68)	n.a.	1.21 (0.81, 1.77)	0.62
Insulin sensitivity index	3.29 ± 1.51	n.a.	4.75 ± 2.41	<0.001
Disposition Index	4.36 (2.12, 8.68)	n.a.	5.21 (3.00, 7.23)	0.37
HOMA-beta	1.48 (1.12, 2.00)	n.a.	1.01 (0.81, 1.83)	0.003
HOMA-IR	2.43 (1.73, 4.65)	n.a.	1.76 (1.48, 3.42)	0.01
HbA1c [mmol/mol]	45.5 ± 11.9	35.1 ± 4.1	37.2 ± 4.4	<0.001
Triglycerides [mmol/L]	1.74 ± 0.84	1.62 ± 1.19	1.41 ± 0.79	0.02
Total cholesterol [mmol/L]	4.38 ± 1.12	5.02 ± 1.12	4.69 ± 0.92	0.47
HDL cholesterol [mmol/L]	1.32 ± 0.35	1.21 ± 0.25	1.20 ± 0.32	0.06
LDL cholesterol [mmol/L]	2.27 ± 0.89	3.15 ± 1.02	2.86 ± 0.89	0.04
ALT [µkat/L]	0.92 (0.74, 1.28)	0.42 (0.37, 0.62)	0.42 (0.34, 0.54)	<0.0001
AST [µkat/L]	0.49 ± 0.34	0.39 ± 0.13	0.37 ± 0.12	0.02
Urea [mmol/L]	5.70 ± 2.64	5.29 ± 1.46	5.60 ± 1.40	0.82
Creatinine [µmol/L]	66.5 ± 14.7	71.7 ± 15.6	74.6 ± 15.2	<0.0001
Albumine [g/L]	40.7 ± 1.9	47.1 ± 5.4	43.0 ± 2.5	<0.0001
Cortisol [nmol/L]	214 ± 181	n.a.	241 ± 97	0.68
TSH [µmol/L]	2.29 ± 1.54	n.a.	1.73 ± 1.13	0.02
Triiodothyronine (T3) [pmol/L]	5.20 ± 0.81	n.a.	5.76 ± 0.52	0.002
Thyroxine (T4) [pmol/L]	15.8 ± 3.5	n.a.	14.5 ± 1.9	0.01
White blood cells [x10^9/L]	9.93 ± 3.86	6.50 ± 1.57	5.92 ± 1.62	<0.0001
Platelets [x10^9/L]	243 ± 105	247 ± 61	207 ± 54	0.09
D-dimer [µg/L]	680 (300, 1320)	520 (315, 890)	255 (210, 530)	<0.0001
Baecke Score	6.5 ± 1.3	7.6 ± 1.4	7.5 ± 1.4	0.001

Normally distributed variables were expressed as mean ± standard deviation and skewed distributed variables as median and interquartile range
P-values for differences between T0 and T6 were calculated with paired T-test for normal distributed variables, with Wilcoxon signed rank test for skewed distributed variables and via Chi-square test for categorical variables.

Supplement Table 2: COVID-19 Related Symptoms: Absolute number of symptom occurrences and percentage representation in the study cohort during baseline (T0) and follow-up visits (T3, T6), n = total number of participants.

Symptoms	T0<br>n = 21*	T3<br>n = 37	T6<br>n = 36
Fatigue	19 (90.48 %)	24 (64.86 %)	12 (33.33 %)
Muscle aiches	5 (23.81 %)	6 (16.22 %)	3 (8.33 %)
Joint pain	1 (4.76 %)	6 (16.22 %)	8 (22.22 %)
Muscle weakness/decondition	19 (90.48 %)	17 (45.95 %)	10 (27.78 %)
Fever	2 (9.52 %)	0 (0.00 %)	0 (0.00 %)
Sweating	4 (19.05 %)	5 (13.51 %)	2 (5.56 %)
Weight loss	10* (66.67 %)	2 (5.41 %)	3 (8.33 %)
Reduced oral intake	10* (66.67 %)	7 (18.92 %)	5 (13.89 %)
Dyspnea	12 (57.14 %)	19 (51.35 %)	7 (19.44 %)
Cough	7 (33.33 %)	4 (10.81 %)	3 (8.33 %)
Chest pain	0 (0.00 %)	5 (13.51 %)	4 (11.11 %)
Palpitation	1 (4.76 %)	5 (13.51 %)	3 (8.33 %)
Depression	2 (9.52 %)	3 (8.11 %)	5 (13.89 %)
Insomnia	9 (42.86 %)	5 (13.51 %)	8 (22.22 %)
PTSD	3 (14.29 %)	0 (0.00 %)	1 (2.78 %)
Loss of smell/taste	5 (23.81 %)	2 (5.41 %)	2 (5.56 %)
Cefalea	2 (9.52 %)	4 (10.81 %)	5 (13.89 %)
Cognitive imparement	1 (10.00 %)**	6 (16.22 %)	6 (16.67 %)
Diarhoe	0 (0.00 %)	2 (5.41 %)	3 (8.33 %)
Hair loss	4 (19.05 %)	17 (45.95 %)	9 (25.00 %)
Skin rash	1 (4.76 %)	1 (2.70 %)	0 (0.00 %)

* Only 21 returned questionnaires were available at T0
** Only 15 participants answered the question about the symptom at T0 (n = 15).

Supplement Table 3: Characteristics of the study population at baseline (T0) and follow-up visit after 6 months (T6) divided by glycemia status (n=26)

	T0			T6		
	Normal glycemia (IFG < 5.6 and IGT < 7.8 mmol/L)	Hyperglycemia (IFG ≥ 5.6 or IGT ≥ 7.8 mmol/L)	p	Normal glycemia at T0 (IFG < 5.6 and IGT < 7.8 mmol/L)	Hyperglycemia at T0 (IFG ≥ 5.6 or IGT ≥ 7.8 mmol/L)	p
n	9	17	-	9	17	
Age [years]	54.5 ± 13.3	61.5 ± 6.8	0.08	54.5 ± 13.3	61.5 ± 6.8	0.08
Sex [n female, %]	3 (33%)	6 (35%)	0.92	3 (33%)	6 (35%)	0.92
BMI [kg/m²]	31.7 ± 4.5	29.8 ± 5.0	0.35	32.9 ± 4.3	31.0 ± 4.7	0.30
Body weight [kg]	93.6 ± 16.9	89.7 ± 14.9	0.55	97.5 ± 17.5	93.2 ± 14.3	0.51
WHR	0.95 ± 0.07	0.94 ± 0.09	0.77	0.94 ± 0.07	0.92 ± 0.09	0.69
Fat mass [%]	25 ± 12	24 ± 9	0.91	30 ± 19	24 ± 9	0.25
Systolic blood pressure [mmHg]	130 ± 11	133 ± 18	0.65	132 ± 17	137 ± 18	0.50
Diastolic blood pressure [mmHg]	83 ± 4	85 ± 8	0.52	83 ± 4	85 ± 11	0.55
First random glucose [mmol/L]	5.9 (5.7, 6.4)	6.7 (6.3, 8.4)	0.02	-	-	-
Fasting glucose [mmol/L]	4.7 ± 0.5	5.7 ± 1.2	0.03	5.0 ± 0.4	5.6 ± 0.6	0.02
Glucose after 2 hours [mmol/L]	6.9 ± 0.7	10.3 ± 2.3	<0.001	6.7 ± 0.8	8.1 ± 2.0	0.05
Fasting insulin [mIU/L]	13.1 ± 8.8	15.1 ± 6.7	0.52	9.3 ± 3.5	12.0 ± 9.4	0.42
Insulin after 2 hours [mIU/L]	103.0 ± 64.5	75.2 ± 30.4	0.14	62.6 ± 41.6	74.6 ± 57.6	0.59
Fasting C-peptide [pmol/L]	940 ± 276	863 ± 275	0.50	513 ± 64	584 ± 264	0.44
C-peptide after 2 hours [pmol/L]	3682 ± 877	3159 ± 704	0.11	2242 ± 619	2349 ± 777	0.72
Fasting NEFA [mmol/L]	0.91 ± 0.25	0.99 ± 0.26	0.46	0.71 ± 0.37	0.80 ± 0.20	0.45
NEFA after 2 hours [mmol/L]	0.51 ± 0.17	0.59 ± 0.22	0.37	0.29 ± 0.13	0.36 ± 0.14	0.26
Fasting glycerol [µmol/L]	250 ± 58	266 ± 80	0.61	152 ± 59	159 ± 41	0.72
Glycerol after 2 hours [µmol/L]	222 ± 44	215 ± 59	0.76	113 ± 29	125 ± 23	0.27
Insulinogenic index	2.78 ± 1.77	1.01 ± 0.51	0.001	1.35 ± 0.64	1.57 ± 2.28	0.78
Insulin sensitivity index	3.47 ± 1.78	3.12 ± 1.23	0.57	4.56 ± 1.27	4.52 ± 2.75	0.97
Disposition Index	8.40 ± 5.42	3.05 ± 1.79	0.002	5.67 ± 1.98	4.45 ± 2.83	0.26
HOMA-beta	1.63 ± 1.07	2.00 ± 0.92	0.36	1.20 ± 0.41	1.60 ± 1.24	0.37
HOMA-IR	2.69 ± 1.87	4.03 ± 2.44	0.17	2.04 ± 0.62	3.20 ± 2.83	0.24
$REE_{Harris\ Benedict}$ [kcal/d]	1776 ± 330	1687 ± 237	0.44	1827 ± 342	1735 ± 289	0.43
REE_0 [kcal/d]	2058 ± 283	2048 ± 359	0.94	1711 ± 329	1784 ± 265	0.54
REE_{120} [kcal/d]	2148 ± 274	2147 ± 341	0.99	1844 ± 250	1966 ± 288	0.29
ΔREE_{120-0} [kcal/d]	90 ± 213	121 ± 179	0.70	133 ± 96	182 ± 125	0.32
$REE_0/REE_{Harris\ Benedict}$ [%]	117 ± 12	122 ± 15	0.45	94 ± 6	103 ± 9	0.01
REE_0/ATM ratio [kcal/kg]	29.9 ± 3.9	30.5 ± 4.1	0.71	24.0 ± 2.6	25.4 ± 2.3	0.18

RQ_0	0.70 ± 0.06	0.70 ± 0.08	0.94	0.76 ± 0.05	0.73 ± 0.04	0.13
RQ_{120}	0.79 ± 0.09	0.80 ± 0.09	0.90	0.79 ± 0.09	0.80 ± 0.09	0.90
ΔRQ_{120-0}	0.09 ± 0.06	0.10 ± 0.08	0.83	0.09 ± 0.06	0.06 ± 0.05	0.20
HbA1c [mmol/mol]	41.4 ± 3.0	50.5 ± 15.7	0.10	35.9 ± 2.4	38.9 ± 4.6	0.08
Triglycerides [mmol/L]	1.66 ± 0.55	1.82 ± 1.04	0.68	1.34 ± 0.55	1.59 ± 0.95	0.48
Total cholesterol [mmol/L]	4.15 ± 0.78	4.48 ± 0.87	0.36	4.64 ± 1.14	4.84 ± 0.82	0.60
HDL cholesterol [mmol/L]	1.36 ± 0.40	1.26 ± 0.32	0.53	1.19 ± 0.20	1.19 ± 0.39	1.00
LDL cholesterol [mmol/L]	2.05 ± 0.74	2.37 ± 0.79	0.33	2.84 ± 1.10	2.94 ± 0.82	0.80
ALT [µkat/L]	1.16 ± 0.45	1.08 ± 0.64	0.74	0.42 ± 0.12	0.48 ± 0.22	0.45
AST [µkat/L]	0.43 ± 0.16	0.52 ± 0.22	0.29	0.31 ± 0.07	0.39 ± 0.11	0.06
Urea [mmol/L]	5.06 ± 3.43	5.87 ± 2.48	0.49	5.30 ± 1.72	6.00 ± 1.23	0.24
Creatinine [µmol/L]	73.8 ± 22.1	66.0 ± 10.2	0.23	76.3 ± 19.2	75.5 ± 14.1	0.90
Albumine [g/L]	40.0 ± 2.1	40.5 ± 1.9	0.52	43.5 ± 3.3	42.8 ± 2.2	0.52
Cortisol [nmol/L]	267 ± 175	254 ± 194	0.87	288 ± 87	229 ± 104	0.15
TSH [µmol/L]	1.58 (1.41, 1.84)	2.08 (1.71, 3.75)	0.05	1.49 (1.23, 1.88)	1.53 (1.41, 1.73)	0.98
Triiodothyronine (T3) [pmol/L]	5.38 ± 0.89	5.19 ± 0.63	0.53	5.67 ± 0.50	5.77 ± 0.55	0.65
Thyroxine (T4) [pmol/L]	17.2 ± 3.4	15.2 ± 3.7	0.20	15.3 ± 1.6	14.2 ± 2.0	0.16
White blood cells [$x10^9$/L]	8.77 ± 2.28	10.12 ± 4.81	0.44	6.46 ± 1.79	5.87 ± 1.62	0.40
Platelets [$x10^9$/L]	226 ± 86	237 ± 126	0.81	222 ± 69	204 ± 52	0.46
D-dimer [µg/L]	850 (340, 1210)	1130 (420, 2140)	0.54	350 (220, 540)	235 (200, 515)	0.34
Baecke Score	5.9 ± 1.0	6.9 ± 1.4	0.08	7.3 ± 1.4	8.7 ± 1.5	0.51

Normally distributed variables were expressed as mean ± standard deviation and skewed distributed variables as median and interquartile range

P-values for differences between normal glycaemic and hyperglycaemic group at T0 and T6, respectively, were calculated with unpaired T-test for normal distributed variables, with Wilcoxon signed rank test for skewed distributed variables and via Chi-square test for categorical variables

Supplement Table 4: Sensitivity Analysis: Baseline characteristics of the study population without excluding participants at baseline (T0) that were lost to follow-up at 6 months (T6) divided by glycemia status (n=37)

	Normal glycemia (IFG < 5.6 and IGT < 7.8 mmol/L)	Hyperglycemia (IFG ≥ 5.6 or IGT ≥ 7.8 mmol/L)	p
n	15	22	-
Age [years]	53.2 ± 11.6	58.4 ± 8.9	0.13
Sex [n female, %]	7 (47%)	7 (32%)	0.36
BMI [kg/m²]	32.2 ± 5.6	29.3 ± 4.4	0.09
Body weight [kg]	94.3 ± 19.2	88.1 ± 14.2	0.27
WHR	0.92 ± 0.08	0.94 ± 0.08	0.58
Fat mass [%]	27 ± 12	24 ± 9	0.38
Systolic blood pressure [mmHg]	127 ± 11	134 ± 18	0.18
Diastolic blood pressure [mmHg]	82 ± 5	85 ± 7	0.20
First random glucose [mmol/L]	6.0 (5.7, 6.2)	6.9 (6.3, 7.5)	0.002
Fasting glucose [mmol/L]	4.7 ± 0.6	5.7 ± 1.1	0.004
Glucose after 2 hours [mmol/L]	6.5 ± 1.0	9.8 ± 2.4	<0.0001
Fasting insulin [mIU/L]	12.7 ± 8.3	14.8 ± 6.7	0.40
Insulin after 2 hours [mIU/L]	87.6 ± 56.2	87.1 ± 47.6	0.97
Fasting C-peptide [pmol/L]	941 ± 391	889 ± 301	0.65
C-peptide after 2 hours [pmol/L]	3476 ± 1074	3324 ± 981	0.66
Fasting NEFA [mmol/L]	0.89 ± 0.24	0.95 ± 0.25	0.46
NEFA after 2 hours [mmol/L]	0.53 ± 0.16	0.57 ± 0.22	0.61
Fasting glycerol [µmol/L]	244 ± 55	256 ± 81	0.65
Glycerol after 2 hours [µmol/L]	214 ± 53	215 ± 68	0.99
Insulinogenic index	2.68 (1.46, 4.06)	0.96 (0.76,1.47)	0.001
Insulin sensitivity index	3.77 ± 1.89	2.99 ± 1.18	0.15
Disposition Index	8.92 (4.94, 16.97)	3.04 (1.59, 4.74)	0.003
HOMA-beta	1.59 ± 1.02	1.96 ± 0.92	0.26
HOMA-IR	2.64 ± 1.84	3.91 ± 2.34	0.09
$REE_{Harris\ Benedict}$ [kcal/d]	1772 ± 361	1694 ± 237	0.44
REE_0 [kcal/d]	2025 ± 326	2053 ± 384	0.82
REE_{120} [kcal/d]	2095 ± 290	2146 ± 351	0.65
ΔREE_{120-0} [kcal/d]	70 ± 229	110 ± 187	0.57
$REE_0/REE_{Harris\ Benedict}$ [%]	116 ± 12	121 ± 15	0.25
REE_0/ATM ratio [kcal/kg]	30.2 ± 4.7	30.9 ± 4.1	0.65
RQ_0	0.72 ± 0.08	0.69 ± 0.08	0.28
RQ_{120}	0.81 ± 0.08	0.79 ± 0.09	0.31
ΔRQ_{120-0}	0.09 ± 0.07	0.09 ± 0.08	0.96
HbA1c [mmol/mol]	41.0 ± 3.9	48.5 ± 14.5	0.06
Triglycerides [mmol/L]	1.62 ± 0.46	1.81 ± 1.02	0.50
Total cholesterol [mmol/L]	4.24 (3.29, 4.74)	4.44 (3.73, 5.61)	0.35
HDL cholesterol [mmol/L]	1.37 ± 0.33	1.29 ± 0.36	0.52
LDL cholesterol [mmol/L]	2.05 ± 0.82	2.41 ± 0.92	0.24
ALT [µkat/L]	1.15 (0.61, 1.38)	0.89 (0.74, 1.12)	0.52
AST [µkat/L]	0.40 ± 0.14	0.56 ± 0.42	0.18
Urea [mmol/L]	5.39 ± 2.84	5.92 ± 2.54	0.55
Creatinine [µmol/L]	69.5 ± 18.6	64.5 ± 11.4	0.32
Albumine [g/L]	40.5 ± 2.1	40.8 ± 1.8	0.60
Cortisol [nmol/L]	196 ± 174	226 ± 189	0.62
TSH [µmol/L]	1.69 (1.38, 2.90)	1.94 (1.50, 2.66)	0.51
Triiodothyronine (T3) [pmol/L]	5.34 ± 0.93	5.10 ± 0.72	0.37
Thyroxine (T4) [pmol/L]	17.3 ± 3.3	14.8 ± 6.7	0.03
White blood cells [$x10^9$/L]	9.58 ± 2.81	10.17 ± 4.49	0.65
Platelets [$x10^9$/L]	256 ± 93	235 ± 115	0.56
D-dimer [µg/L]	430 (260, 1000)	680 (310, 2140)	0.25
Baecke score	6.1 ± 1.2	6.8 ± 1.2	0.10

Normally distributed variables were expressed as mean ± standard deviation and skewed distributed variables as median and interquartile range

P-values for differences between normal glycaemic and hyperglycaemic group were calculated with unpaired T-test for normal distributed variables, with Wilcoxon signed rank test for skewed distributed variables and via Chi-square test for categorical variables

Supplement Table 5: Sensitivity Analysis: Clinical metabolic variables at baseline (T0) and follow-up visit after 3 (T3) and 6 months (T6) and the mean difference plus 95% CI between T0 and T6 in the full data-set population

	T0		T3		T6		ΔT6-T0		
	n	mean ± SD	n	mean ± SD	n	mean ± SD	n	mean difference (95% CI)	*p*
$REE_{Harris\ Benedict}$ [kcal/d]	37	1726 ± 291	32	1759 ± 276	31	1782 ± 272	31	**49 (34, 65)**	<0.0001
REE_0 [kcal/d]	37	2042 ± 357	32	1796 ± 283	31	1756 ± 280	31	**-302 (-393, -212)**	<0.0001
REE_{120} [kcal/d]	36	2125 ± 324		n.a.	31	1921 ± 264	30	**-229 (-330, -128)**	<0.0001
ΔREE_{120-0} [kcal/d]	36	93 ± 203		n.a.	31	165 ± 113	30	71 (-6, 148)	0.07
$REE_0/REE_{Harris\ Benedict}$ [%]	37	119 ± 14	32	102 ± 9	31	99 ± 10	31	**-20 (-25, -16)**	<0.0001
REE_0/ATH [kcal/kg]	37	30.6 ± 4.3	31	25.4 ± 2.4	31	24.8 ± 2.6	31	**-5.5 (-6.9, -4.1)**	<0.0001
RQ_0	37	0.71 ± 0.08	32	0.76 ± 0.06	31	0.75 ± 0.04	31	**0.04 (0.01, 0.07)**	<0.01
RQ_{120}	36	0.80 ± 0.08		n.a.	31	0.82 ± 0.05	30	0.02 (-0.01, 0.06)	0.19
ΔRQ_{120-0}	36	0.09 ± 0.08		n.a.	31	0.07 ± 0.05	30	-0.02 (-0.05, 0.01)	0.23

Data are shown as mean ± SD. Differences between T6 and T0 are shown as mean difference ± 95% CI. P-values were derived from paired T-test.
ATM, active tissue mass; CI, confidence interval; n.a., not applicable; REE, resting energy expenditure; RQ, respiratory quotient; SD, standard deviation; T0, baseline visit; T3, visit after three months; T6, visit after six month

Supplement Table 6: Sensitivity Analysis: Outcome indices at baseline (T0) and follow-up visit after 6 months (T6) and the mean differences or median of differences plus 95% CI between these visits in the normal glycemic and hyperglycemic groups in the full data-set

	Normal glycemia		Hyperglycemia	Adjusted group differences	
	**T0 (n=37)**				
	mean ± SD / median (IQR)*		mean ± SD / median (IQR)*	mean difference (95% CI) / median of difference (95% CI)*	*p*
Insulinogenic index*	2.68 (1.46, 4.06)		0.96 (0.76, 1.47)	**-1.32 (-2.43, -0.21)**	0.02
Insulin sensitivity index	3.77 ± 1.89		2.99 ± 1.18	**-1.23 (-2.29, -0.17)**	0.02
Disposition Index*	8.92 (4.94, 16.97)		3.04 (1.59, 4.74)	-4.58 (-10.42, 1.27)	0.12
HOMA-beta	1.59 ± 1.02		1.96 ± 0.92	**0.65 (0.00, 1.29)**	0.05
HOMA-IR	2.64 ± 1.84		3.91 ± 2.34	**1.85 (0.39, 3.30)**	0.01
	**T6 (n=31)**				
Insulinogenic index*	1.50 (0.99, 2.26)		1.15 (0.60, 1.55)	-0.48 (-4.63, 3.67)	0.82
Insulin sensitivity index	5.03 ± 2.17		4.56 ± 2.59	-0.71 (-2.52, 1.11)	0.43
Disposition Index*	5.96 (4.80, 8.42)		4.32 (2.96, 6.89)	-1.57 (-6.01, 2.88)	0.48
HOMA-beta	1.18 ± 0.48		1.53 ± 1.18	0.48 (-0.27, 1.23)	0.20
HOMA-IR	2.04 ± 0.81		3.05 ± 2.70	1.25 (-0.45, 2.94)	0.14
	**ΔT6-T0 (n=31)**				
	Normal glycemia			Hyperglycemia	
	mean difference (95% CI) / median of difference (95% CI)*			mean difference (95% CI) / median of difference (95% CI)*	
Insulinogenic index*	-0.85 (-2.59, 4.75)	0.41		-0.02 (-0.16, 0.37)	0.73
Insulin sensitivity index	+0.98 (-0.24, 2.21)	0.10		**+1.93 (0.71, 3.15)**	0.004
Disposition Index*	0.67 (-7.18, 21.69)	0.83		1.71 (-0.17, 2.92)	0.21
HOMA-beta	-0.32 (-0.83, 0.20)	0.20		**-0.58 (-1.02, -0.15)**	0.01
HOMA-IR	-0.44 (-1.35, 0.47)	0.31		**-1.22 (-2.15, -0.28)**	0.01

Data are shown as mean ± SD. Differences between the normal glycaemic and hyperglycaemic group are shown as mean difference ± 95% CI adjusted for age, sex and BMI. P-values were derived from unpaired T-test. Differences over time between T6 and T0 were derived from paired T-test

CI, confidence interval; HOMA-beta, homeostasis model assessment of beta cell function; HOMA-IR, homeostasis model assessment of insulin resistance; SD, standard deviation; T0, baseline visit; T6, visit after six month; ΔT6-T0, change between T6 and T0;

* skewed distributed variables are shown as median and IQR and median of difference and 95% CI

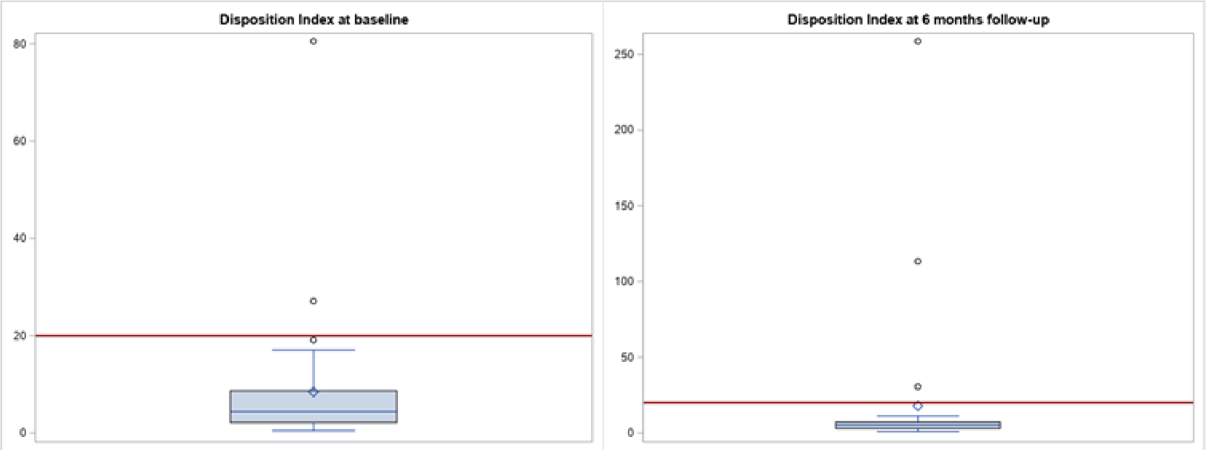

Excluded extreme outliers were: patients with a disposition index of 27.1 and 80.6 at baseline and with values of 30.6, 113.4 and 258.7 at follow-up after 6 months

Supplement Figure 1: Distribution of the disposition index (DI) at baseline and after 6 months follow-up: boxplots are showing extreme outliers that were excluded from the main analysis.

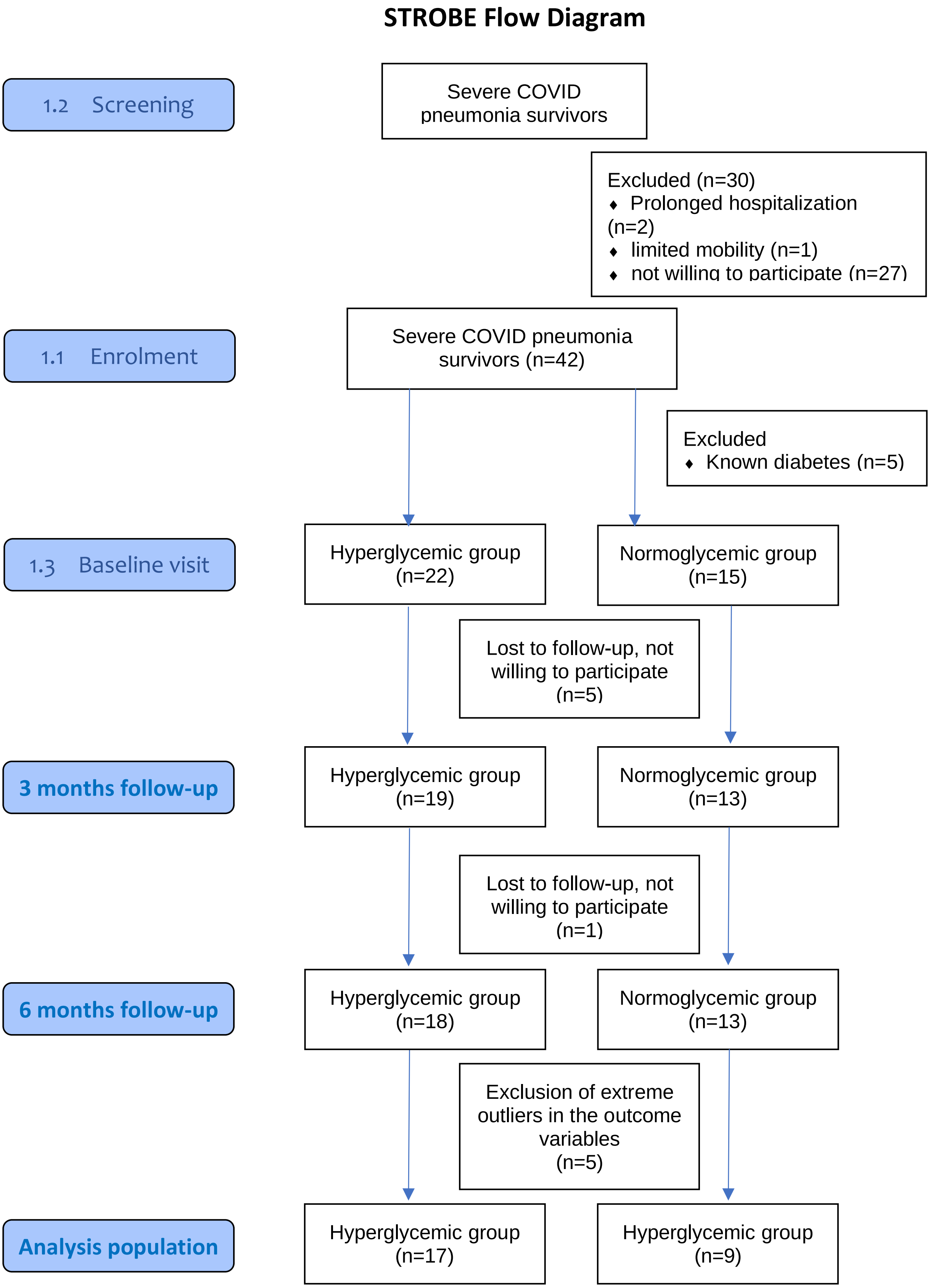

Supplement Figure 2: STROBE flow diagram. From 37 subjects enrolled and examined at the T0 5 patients were considered as outliers in the major outcome variables and were not included in the final analysis.

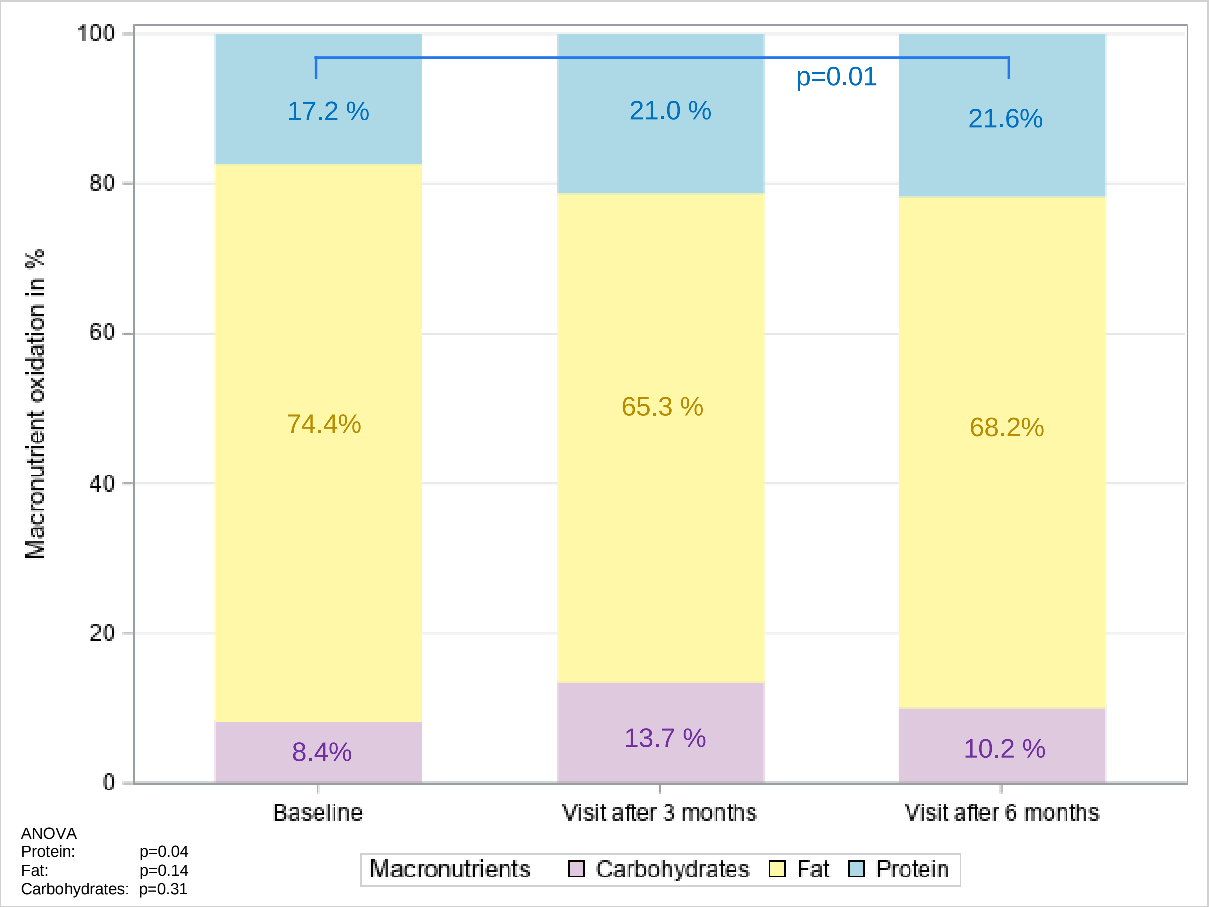

Differences between macronutrient oxidation at baseline and follow-up after 3 and 6 months were tested via repeated measures ANOVA including p-values for differences between two or all three time points;
*p-values <0.05 indicating differences in time for macronutrient oxidation between two time points for protein between baseline and visit after 6 months (p= 0.01)

Supplement Figure 1: Oxidation of carbohydrates, fat and protein expressed as a share from 100% at baseline (n=37) and follow-up after 3 months (n=32) and 6 months (n=31) in the full data-set
